# Drug resistance: from bacteria to cancer

**DOI:** 10.1186/s43556-021-00041-4

**Published:** 2021-09-10

**Authors:** Harsh Patel, Zhuo-Xun Wu, Yanglu Chen, Letao Bo, Zhe-Sheng Chen

**Affiliations:** 1 Department of Pharmaceutical Sciences, College of Pharmacy and Health Sciences, St. John’s University, Queens, New York, NY 11439 USA; 2 Columbia University Vagelos College of Physicians and Surgeons, New York, NY 10032 USA

**Keywords:** Drug resistance, Efflux pumps, Chemotherapy, ATP-binding cassette transporters, Integrons

## Abstract

The phenomenon of drug resistance has been a hindrance to therapeutic medicine since the late 1940s. There is a plethora of factors and mechanisms contributing to progression of drug resistance. From prokaryotes to complex cancers, drug resistance is a prevailing issue in clinical medicine. Although there are numerous factors causing and influencing the phenomenon of drug resistance, cellular transporters contribute to a noticeable majority. Efflux transporters form a huge family of proteins and are found in a vast number of species spanning from prokaryotes to complex organisms such as humans. During the last couple of decades, various approaches in analyses of biochemistry and pharmacology of transporters have led us to understand much more about drug resistance. In this review, we have discussed the structure, function, potential causes, and mechanisms of multidrug resistance in bacteria as well as cancers.

## Introduction

Paul Ehrlich introduced us to the terms- “chemotherapy” and “the Magic bullet”, where he explained the idea of a therapy that would kill the microbe and leave the affected individual harmless. He then went on to develop a drug against *Treponema pallidum* (a bacterium that causes syphilis) which was the only available treatment option against bacterial infections for the next 20 years [1]. Sir Alexander Fleming discovered the antibiotic penicillin from the fungi *Penicillium notatum*, which changed the course of medical science. The success of this discovery was seen in World War II as it helped millions of soldiers who used antibiotics to fight against the “invisible enemy” [2]. The history of medicine catalogs numerous similar episodes that involves antibiotics. It was not until recently that we have experienced the phenomenon of drug resistance. As a fact of the matter, World Health Organization (WHO) said that drug resistance is one of the three serious challenges of public health of the twenty-first century [3].

Bacterial antibiotic resistance is a continuing and increasing concern in clinical science. Owing to a shorter lifespan, bacteria are more susceptible to genetic variation and evolution than other eukaryotic organisms. *Escherichia coli* has a unique identity in the microbial world as it is also an essential gut bacterium as well as a harmful pathogen. The inquilinity of *E. coli* with various mammals allows it to cause infections ranging from mild diarrhea to severe colitis. *E. coli* exhibits a wide range of resistance against drugs such as β-lactams, ampicillin, cephalosporin, quinolones, aminoglycosides, and several other antibiotics [4]. A similar resistance spectrum is shown by methicillin resistant *Staphylococcus aureus* (MRSA)*, Mycobacterium tuberculosis*, *Pseudomonas aeruginosa*, *Acinetobacter baumanii* and a few other gram-negative bacteria. Multidrug resistance is mainly observed in bacteria due to a build-up of drug-resistance plasmids (R) or transposons that encode drug resistant genes, by drug efflux pumps or by both of these mechanisms [5]. Moreover, the target-protein modification that results in making the bacteria less susceptible to the drug can also aid in drug resistance. For example, the *erm* gene methylates the adenine at position 2058 of the 50s rRNA resulting in drug resistant bacteria [6]. Additionally, the drug resistance mechanism against naturally originating antibiotics is generally observed due to enzymatic inactivation of the drug, of which, enzymatic phosphorylation, adenylation, acetylation and hydrolysis lead to drug resistance. One of the most intriguing causes of drug resistance is the horizontal gene transfer from the same or different species of bacteria [7]. Horizontal gene transfer (HGT) is a process of transfer of a part of genetic material to a cell that is not its progeny, and it can be attained by the activity of either plasmids or transposons.

Chemotherapy has been one of the leading therapeutic regimens for cancer patients. However, clinical oncologists observe that patients develop cancer resistance against drugs that they have never been exposed to. This essentially deteriorates the effectivity of chemotherapy. In many research studies, cells in vitro are exposed to such chemotherapeutic drugs and a similar pattern of drug resistance is seen, just like in vivo*.* This phenomenon of resistance to multiple drugs is known as multidrug resistance (MDR), and it may be passed on to the daughter cells from the parent cancer cells. Alterations or mutations in MDR proteins [mostly adenosine triphosphate (ATP)-binding cassette (ABC) transporters] is a common mechanism for development of drug-resistance. This affects the normal cellular functions like signal transduction, uptake of extracellular materials, cellular transport and excretion, secretion of proteins or hormones, lipid transport and prevention of harmful xenobiotics, to name a few (Fig. 2). However, there is not a clear idea as to how the mechanism of MDR in vivo is responsible. Research from the past few years have concluded that P-glycoprotein (P-gp/ABCB1) is one of the major causes of MDR in cancers; although, studies about different transporters are in progress. The dynamics of P-gp are such that it can recognize multiple substrates. In this review, a few members of the ABC superfamily of transporters are described.

## Antibacterial drug-resistance mechanisms

The bacteria develop drug resistance via multiple mechanisms. This can be either genetic or mechanistic or both. These mechanisms include acquired resistance through horizontal gene transfer or through xenobiotic transfer, protein modification of target or receptor, through drug efflux pumps or by preventing drug influx.

**Fig. 1:**
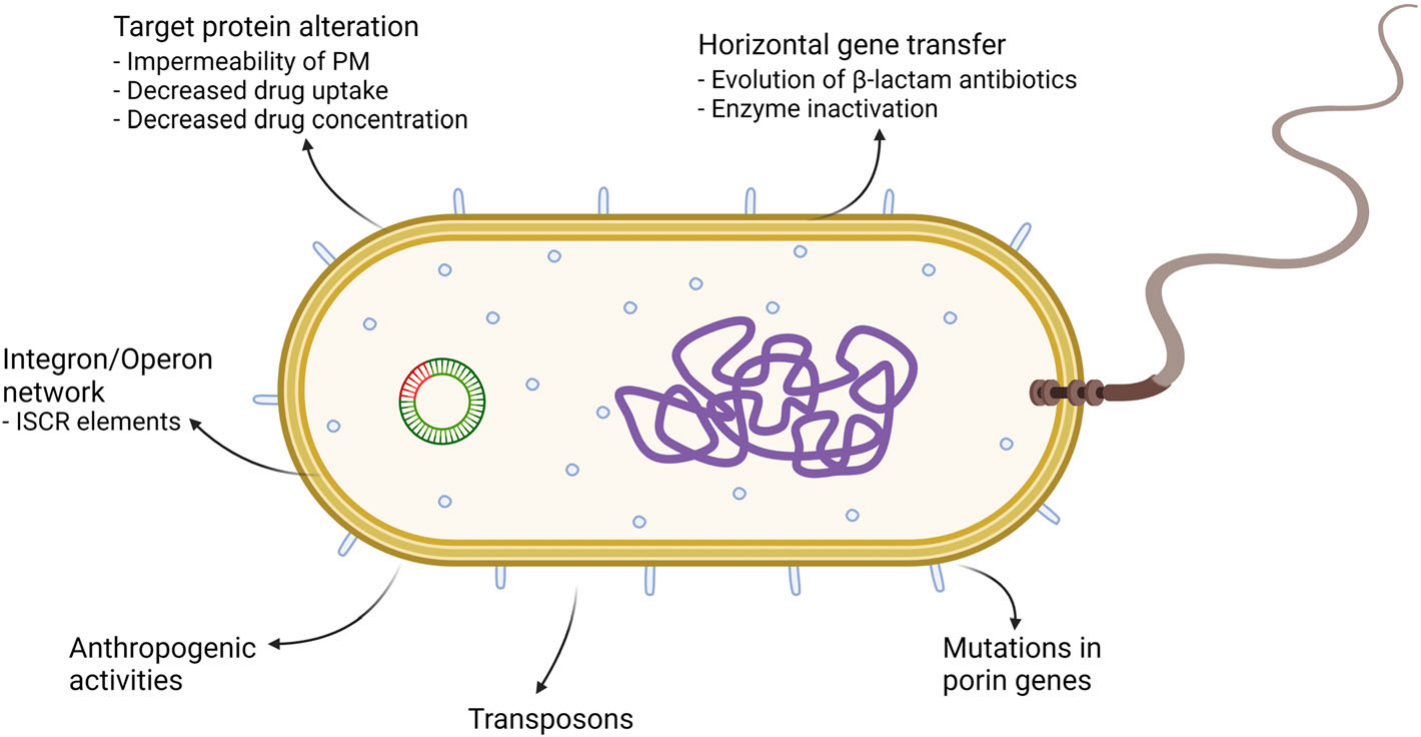
Drug resistance in bacteria. *Target protein alteration.* Certain modifications lead to impermeability of the cell membrane and thus decrease drug uptake. Target modification leads to a demoted drug binding. *Integron Operon network.* Integrons help insert a resistance gene at a pre-decided site downstream of a promoter (Example- *Tn21*). *Anthropogenic activities.* Release of toxic chemicals into the environment provides a selection and survival pressure which leads to variation and ultimately, evolution. *Horizontal gene transfer.* Transfer of genes from other species or from same species, but not parental cells is called HGT. Evolution of β-lactam antibiotic resistance genes is one of the results of HGT. *Transposons.* “Jumping genes” produces enzymes that aid in HGT. *Mutation in porin genes.* It can lead to decreased drug influx or increased drug efflux with the help of ion motive force, as compared to ATP hydrolysis by transporters

Figure 1 gives a brief representation of drug resistance mechanisms observed in bacteria. Transposons or “jumping genes” produce enzymes that facilitate the movement of genes to another locus of deoxyribonucleic acid (DNA) in either same genome or in another organism. Moreover, for drug resistance, an integral factor other than evolution is the acquisition of genes that confers antibiotic resistance, especially via HGT. It can cause large-scale changes in a bacterial genome without causing mutations and thus it is a highly effective drug-resistance mechanism [8]. Furthermore, the evolution of resistance to β-lactam antibiotics is a consequence of HGT [9]. Mutations in genes that encode for target proteins may lead to drug resistance as the drug can no longer bind to the target protein. Most instances of drug resistance arise from genes present on R plasmids. Moreover, these genes are transferred to a vulnerable bacterium in a distinct conjugation episode. At the time of discovery of R plasmids, it was found that many of these resistance genes against common antibiotics such as tetracycline, chloramphenicol, sulfonamides, and aminoglycosides were already present. However, their discovery came to light just recently as a consequence of the sequencing studies [5]. The recent discovery of integrons has also revealed a gene encoding an integrase enzyme that helps insert a resistance gene at a pre-decided site downstream of a promoter. The integron has a unique 59 bp 3′-sequence tag and the resistance gene gets marked by the tag as soon as it is integrated. After this, the gene becomes integrated into another integron, conceivably containing a completely diverse batch of resistance genes (it may contain up to eight resistance genes). It is thought that this insertion into integrons and organization into distinct operons is how resistance genes obtain high mobility. This operon will conclusively have the same transcription direction under a strong promoter which is provided by the integron framework. *Tn21* is one such example of large, complex, and multiple composite transposons which consists of resistance genes against mercury, sulfonamide (*sul1*) and aminoglycoside (*aadA1*) [10–12]. Many integrons also haul enzymatic machinery to transpose the complete integron framework to other loci in the genome [13]. It was not until recently that various integrons were discovered to be associated with a downstream structure known as insertion sequences – common region(s) element (ISCR). The ISCR element mainly functions as a recruiter and a transporter to the integron framework, which results in assembly of more resistance genes [14]. Anthropogenic activities contribute a reasonable amount in conferring drug resistance to bacteria. The amount of toxic wastes, chemicals, antibiotics, metals, intermediates, and numerous xenobiotics released into the environment is immeasurable; and it brings upon ceaseless selection pressure and the maintenance of genome to populations of resistant strains in almost all the environments.

Mutations in porin genes can sometimes lead to drug efflux outside the cell. In addition, drug efflux pumps extrude drug molecules outside the cells. The majority of the activity of drug efflux pumps in bacteria occurs through the activity of ion motive force; however, many pumps use ATP hydrolysis as a driving factor as well. Some of the major drug efflux transporter families are major facilitator superfamily (MFS), small multidrug resistance (SMR) family, resistance/nodulation/division (RND) superfamily, multi antimicrobial extrusion (MATE) family, and ATP-binding cassette transporters (ABC) superfamily.

### Bacterial efflux pumps

In the last few decades, various bacterial drug transporters were discovered and classified (Table 1). Prokaryotic efflux transporters are often referred to as half transporters. To form a functional unit, these transporters either homodimerize with themselves or heterodimerize with other transporters [48]. The normal function of a conventional MDR protein is to uptake and secrete a wide range of substrate(s) including both small molecules such as amino acids, xenobiotics, vitamins, sugars; and complex polymers such as oligopeptides, proteins, and polysaccharides. MDR proteins have an extensive uptake spectrum, allowing them to participate in a variety of cellular processes such as uptake of nutrients, cellular excretion of waste and debris, xenobiotic protection, immunity against foreign bodies, bacterial virulence, osmosis, lipid transport, and biogenesis [55–57]. The primary structure contains four domains: two nucleotide binding domains (NBDs) and two transmembrane domains (TMDs), all of which are encoded by four independent genes or by a blend of the genes or by one single gene that encodes the entire transporter [58]. Along with these four domains, there may be an extracellular substrate binding domain that transfers the substrate to the permease domain [59, 60].

**Table 1:** Bacterial efflux transporters

Name/ Gene name	Other names	Organism	Family/Sub-family	Polypeptide chain length (aa)	Confers resistance to	References
*qacA*	QacA	*Staphylococcus aureus*	DHA2 family (MFS)	514	Benzalkonium chloride, Cetyl-trimethyl ammonium bromide, Ethidium bromide, Chlorhexidine, Pentamidine isethionate	[15, 16]
*norA*	–	*Staphylococcus aureus*	TCR family (MFS)	388	Fluoroquinolones	[17]
*emrB*	–	*Escherichia coli*	EmrA family (MFS)	512	2,4-dinitrophenol, Nalidixic acid, CCCP, thiolactomycin, m-chlorophenylhydrazone	[15, 18, 19]
*mdfA*	cmlA, cmr	*Escherichia coli*	MdfA family (MFS)	410	Ethidium bromide, Tetraphenylphosphonium, Rhodamine, Daunomycin, Benzalkonium, Rifampicin, Tetracycline, Puromycin, Chloramphenicol, Erythromycin, Fluoroquinolones, extreme alkaline pH resistance	[20–25]
*setA*	yabM	*Escherichia coli*	SET family (MFS)	392	Sugar efflux, Sugar detoxification (non-metabolizable)	[26]
*sugE*	–	*Escherichia coli*	SMR family (DMT)	105	Guanidinium, Cetylpyridinium, Cetyldimethylethyl ammonium, Cetrimide cations	[27–29]
*emrE*	mvrC	*Escherichia coli*	SMR family (DMT)	110	DDAC, Ethidium, Methyl viologen, Acriflavine, Tetraphenylphosphonium, Benzalkonium, Propidium, Dequalinium, Streptomycin, Tobramycin	[30–32]
*qacE*	–	*Klebsiella pneumoniae*	SMR family (DMT)	110	Quaternary ammonium compounds	[33]
*qacC*	QacSau	*Staphylococcus aureus*	SMR family (DMT)	107	Quaternary ammonium compounds, Ethidium bromide	[33]
*yvdS*	–	*Bacillus subtilis*	SMR family (DMT)	114		[33]
*acrB*	acrE, AcrAB-TolC	*Escherichia coli*	RND family	1049	Tetracycline, Puromycin, Chloramphenicol, Erythromycin, Rifampicin, Fusidic acid, Acriflavine, Bile salts, Cephalosporins, Crystal violet, Ethidium bromide, Fluoroquinolones, SDS, Triclosan, Triton X-100	[34–37]
*mmpL7*	–	*Mycobacterium tuberculosis*	MmpL sub-family (RND)	920	Phthiocerol dimycocerosate, Isoniazid	[38–40]
*czcA*	–	*Ralstonia metallidurans*	RND family	1063	CZC- Cobalt, Zinc and Cadmium resistance	[41]
*mexB*	–	*Pseudomonas aeruginosa*	RND family	1046	tetracycline, chloramphenicol, ciprofloxacin, streptonigrin, dipyridyl	[42]
*norM*	vcmA	*Vibrio cholerae*	MATE family	457	Norfloxacin, Ciprofloxacin, Ofloxacin, Daunomycin, Doxorubicin, Streptomycin, Kanamycin, Ethidium bromide, Acriflavine	[43–45]
*yeeO*	–	*Escherichia coli*	MATE Family	547	Exports peptides- pepA, pepB, pepD, pepN and flavins- FMN, FAD	[46, 47]
*lmrA*	–	*Lactococcus lactis*	LmrA family (ABC)	590	Various antibiotics	[48, 49]
*bmrA*	yvcC	*Bacillus subtilis*	ABC superfamily	589	Hoechst-33,342, Ethidium bromide, Doxorubicin	[26, 50]
*macB*	–	*Escherichia coli*	Macrolide Exporter family (ABC)	648	Macrolides	[51–53]
*msrA*	–	*Staphylococcus epidermidis*	ABC superfamily	488	Erythromycin, B-streptogramins	[26]
*drrAB*	–	*Streptomyces peucetius*	Drug exporter 1 family	330	Daunorubicin, Doxorubicin	[26, 54]

*CCCP* Carbonyl cyanide m-chlorophenylhydrazine, *DDAC* Dodecyl dimethyl ammonium chloride, *SDS* Sodium dodecyl sulfate, *FMN* Flavin mononucleotide, *FAD* Flavin adenine dinucleotide

MFS superfamily consists of numerous drug efflux transporters which catalyze uniport, symport (solute:cation), antiport (solute:solute) or a combination of these processes, although, most of the members probably operate via H^+^ antiport. Some of the members contain 14 transmembrane segments (TMS) and others contain 12 TMS [26]. QacA and QacB fall into the category of MFS superfamily with 14 TMS and contains several acidic amino acid residues. These pumps mainly extrude biocides and dyes such as benzalkonium chloride, cetyl trimethyl ammonium bromide and ethidium bromide. QacA additionally transports out dicationic biocides such as chlorhexidine and pentamidine isethionate. The difference between these two transporters lies in the TMS10 of QacA, which aids in removal of dicationic compounds [15]. Another example of MDR transporter is EmrB of *E. coli,* which confers resistance against carbonyl cyanide m-chlorophenylhydrazone and antibiotics such as nalidixic acid and thiolactomycin. EmrA is a periplasmic adapter protein that connects the pump to TolC, an outer membrane channel that helps extrude the drugs directly outside the cell. EmrA and EmrB both are encoded by a chromosomal gene [15, 18]. MFS pumps containing 12 TMS includes pumps like NorA, NorB, NorC, LmrP, LmrCD, MdfA, AcrB, and many more. NorA is resistant to fluoroquinolones in *Staphylococcus aureus,* other cationic dyes and cationic inhibitors (Table 1) [17]. NorB and NorC are two other homologs of NorA in *S. aureus* that produces a similar phenotype [61]. However, all the three pumps are inhibited by reserpine [62]. *Lactococcus lactis* possesses LmrP, a secondary transporter of MFS family [63] that acts as a “vacuum cleaner” of the cell membrane and pumps out cationic dyes, daunomycin, tetracycline and macrolides [64]. MdfA from *E. coli* extrudes cationic dyes, chloramphenicol, fluoroquinolones when overexpressed in mutants lacking a constitutive RND pump called AcrB [20].

The SMR family proteins are hydrophobic in nature, with four TMSs, consist of some of the smallest transporters and are a part of drug/metabolite transporter (DMT) superfamily. These are cation-specific multidrug efflux pumps and the model example is EmrE of *E. coli* (about 110 amino acids in length) [30]. Moreover, the SMR family of transporters were first found in *S. aureus* plasmids and later, were also found in gram-negative bacteria. They transport quaternary ammonium biocides such as dodecyl dimethyl ammonium chloride (DDAC) or ethidium bromide [27, 30]. Some of the well-studied transporters include EmrE, QacC, QacE, SugE (Table 1) [27].

The RND superfamily is very well studied as transporters from this family play a vital role of drug resistance in gram-negative bacteria. A general structure for the members can be demarcated as a chain consisting of one TMS at the N-terminal linked to a comparatively hefty extracytoplasmic domain followed by six TMSs linked to another extracytoplasmic domain and finally five TMSs at the C- terminal. These transporters get linked with two other classes of proteins- TolC of *E. coli,* one of the outer membrane factor (OMF) family members [65]; and AcrA of *E. coli*, a periplasmic adapter protein of the membrane fusion protein (MFP) family [66]. This network of proteins allows them to directly transport the drugs out into the medium instead of the periplasmic space. Once transported outside, the drugs will need to tread through the outer membrane barrier which is majorly made of lipopolysaccharide, in order to enter the cell. However, this complex works harmoniously with the outer membrane and effectively prevents this entry of the drug [67]. Some of the RND family pumps exhibit a wide range of substrate specificity, for example, AcrB of *E. coli* can not only pump out lipophilic antibiotics but also dyes, detergents and solvents such as acriflavine, bile salts, chloramphenicol, cephalosporins, crystal violet, ethidium bromide, fluoroquinolones, sodium dodecyl sulphate, tetracyclines, triclosan, Triton X-100, etc. [34, 35]. AcrD, a homolog of AcrB can pump out aminoglycosides in *E. coli*. The original function of AcrB seems to be transport of bile salts as it has highest affinity to bile salts [68]. In nosocomial infections of *Pseudomonas aeruginosa*, increasing fluoroquinolone resistance is observed with the help of MFP family of pumps along with probably an OMF component [69]. In *Mycobacterium tuberculosis,* MmpL7 pump extrudes a complex, non-polar lipid- phthiocerol dimycocerosate [38].

The MATE family of transporters include NorM of *Vibrio parahaemolyticus*, which contains 12 transmembrane helices [43]. The members are about 450 aminoacyls long and they operate by an uncommon mechanism of drug: Na^+^ antiport. It extrudes fluoroquinolones and ethidium and in turn, influxes Na^+^ ions [26, 44].

ABC transporters have a limited role in bacterial drug resistance [70]. Both uptake and drug efflux systems have been found in this superfamily that uses energy from ATP hydrolysis, and usually both systems are localized together. The members of this superfamily contain at least two integral membrane domains (~membrane spanning domains (MSD)) and two cytoplasmic domains (~NBD), which can be found as homodimers or heterodimers. In Gram-negative bacteria, the uptake domains are found in periplasm while in Gram-positive bacteria, they are either present as lipoproteins on the outer surface of the cell membrane, or as cell-surface associated proteins [26]. It is important to note that many ABC transporters in bacteria have shown a homology to human ABC transporters. However, the uptake systems are not observed in mammalian ABC transporters. For instance, LmrA of *L. lactis* is homologous to a half of the P-gp protein of mammalian origin [48, 49]. Biochemical studies showed that the drug resistance mechanism is mainly a combination of drug extrusion and ATP hydrolysis [71]. Other examples of ABC transporters in bacteria include BmrA in *Bacillus subtilis* [72, 73] and MacB in *E. coli.* Interestingly, MacB is expressed with a periplasmic adapter protein MacA (in a similar fashion to AcrA of MFP family) which helps in providing resistance to macrolides when overexpressed [53].

## Mammalian MDR proteins

According to the sequence homology and domain organization, there are seven ABC transporter subfamilies [74, 75] named from “A” to “G”, i.e. ABCA to ABCG, into which 49 ABC transporters have been classified [76]. The general structure of these transporters is comparable to those found in bacteria. Mammalian MDR proteins contain at least two NBDs (cytoplasmic) and TMDs (also called membrane-spanning domains – MSDs; analogous to TMS in bacteria). The cytoplasmic NBD has a similar function i.e., serving as a source of energy to produce hydrolyzed ATP, whereas the TMD is very heterogeneous in function and dynamic in nature and its functions support the binding of a drug, transport channel, dimerization/oligomerization, and trafficking [77]. This heterogeneity allows the transporter to recognize a wide range of substrates and use ATP as an energy source, irrespective of the established concentration gradient [78]. Recent updates confirm that ATP provides energy for substrate transport as well as for continuous conformation changes that allow the transporters to identify a range of substrates [79].

The cellular levels of ions, lipids, hormones, xenobiotics, and other small molecules are regulated by ABC transporters by hauling them in and out across the plasma membrane. Thus, contributing a considerable role in physiological aspects like regulating the organelles (mitochondria, lysosome, endoplasmic reticulum (ER), Golgi apparatus) [80–83]. Transporters such as P-glycoprotein (P-gp/ABCB1), multidrug resistance protein 1 (MRP1/ABCC1) and breast cancer resistance protein (BCRP/ABCG2) transports substances across the cell membrane, thereby conferring excretory and defensive physiological activities. At the blood-brain barrier, blood-testis barrier and blood-placental barrier, the entry of foreign, exogenous molecules is inhibited [84, 85]. Notably, the normal functions of ABC transporters also have a significant effect on pharmacokinetics of drugs: absorption, distribution, metabolism, excretion, and toxicity.

### Genetic and mechanical aspects of MDR

What happens if something goes wrong genetically or mechanistically? A germline mutation can lead to a loss-of-function in a single ABC transporter and can be associated to diseases other than cancer like cystic fibrosis, pseudoxanthoma elasticum, Stargardt macular degeneration, Tangier disease, sitosterolaemia and harlequin ichthyosis [86]. However, these genetic anomalies can be rehabilitated by mRNA stabilization, ribosomal readthrough, correction of folding and trafficking errors, allosteric activation, modulation of protein interactions, regulation of post translational modifications, improving of protein degradation pathways and initiation of other compensatory mechanisms [87–95].

**Fig. 2:**
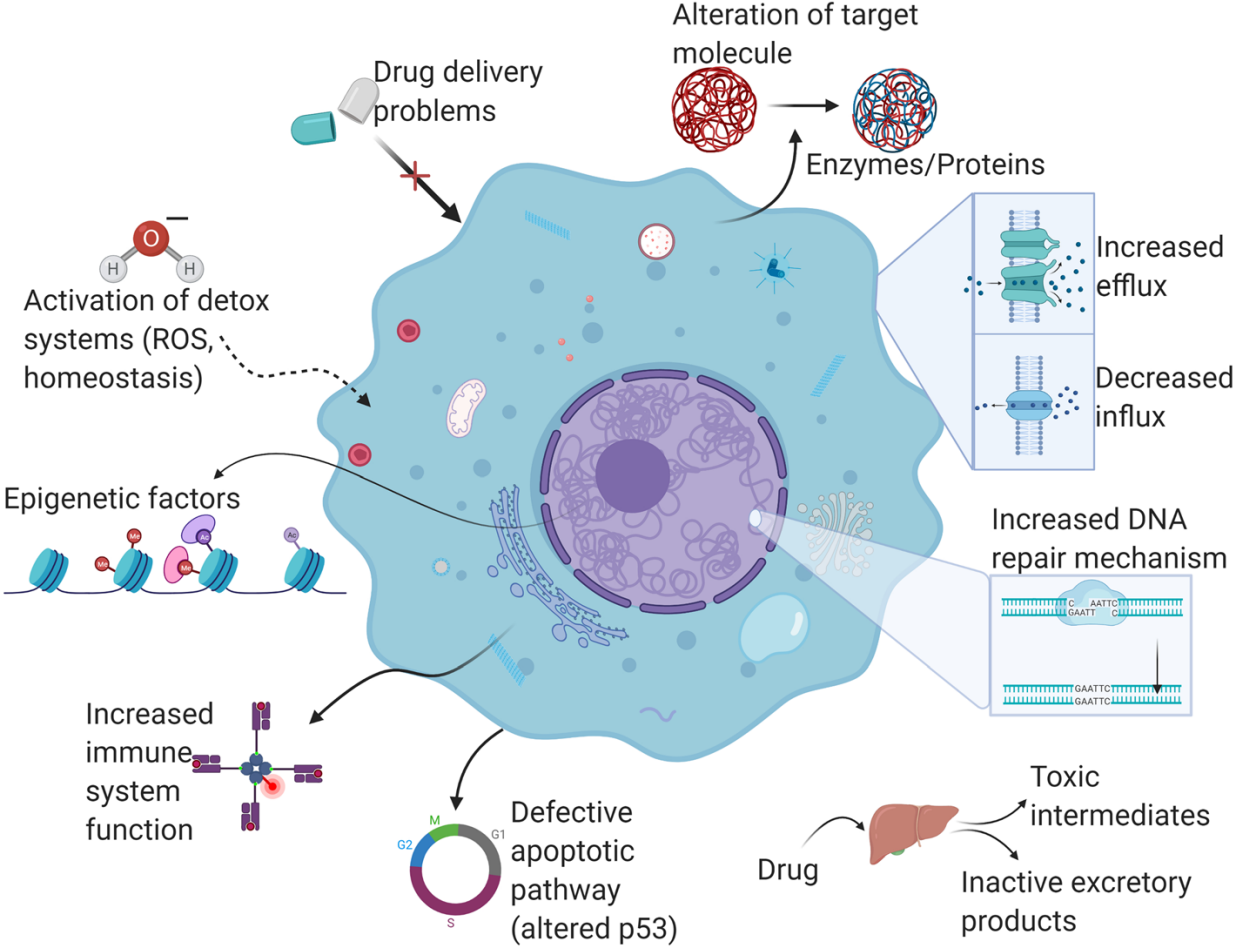
Mechanisms by which drug resistance is conferred in cancer. (In clockwise manner starting with defective apoptotic pathway) (1) the apoptotic pathway (p53 pathway) might be defective, which leads to various downstream resistance mechanisms like upregulation of Nrf2 expression, MGM2 upregulation, increased cell proliferation, etc.; (2) drug resistance is seen when there is increased or defective immune system function where hypersensitivity is observed; (3) a plethora of epigenetic factors play roles in conferring drug resistance in cancer; (4) when the detox systems (ROS, homeostasis) are activated, there is a detox of the drug from the cancer cell; (5) drug delivery problems: too low concentration or higher molecule size than required; (6) drug alteration by intrinsic enzymes or other proteins; (7) transporters of the cell membrane exhibit increased drug efflux or decreased influx; (8) even if the drug enters the cell and affects the genetic machinery, there is an increased intrinsic DNA repair mechanisms; (9) drug resistance is also seen during increased metabolism in liver

Resistance to anti-cancer drugs can be roughly described as complications in drug delivery to tumor cells or problems of impaired drug sensitivity within the cancer cells themselves as a result of genetic/epigenetic alteration. Complications in drug delivery can arise from poor absorption of oral drugs, elevated metabolism, or increased excretion. As a result, there will be lower level of drug in the blood. This means that a proper amount of drug is not reaching the tumor mass (Fig. 2) [96, 97]. Furthermore, irregular pressure gradient along with tumor vasculature was also reported to affect drug delivery [98]. Besides these factors, tumor geometry and composition of the extracellular matrix have been observed to participate in drug resistance [96, 99, 100]. Drug resistance is again observed when the cellular target of a drug is altered in conformation or geometry; or if there is increased DNA maintenance activity in tumor cells. The phenomenon of cross resistance to other structurally and mechanistically irrelevant drugs, after resistance to a single drug is attained, is categorized as MDR [101]. Drug resistance is descried by decreased drug uptake. The transporters and carrier molecules that are used for nutrient uptake are abused as “ferry” molecules by water-soluble drugs, or by the process of endocytosis. This is illustrated by drugs such as methotrexate, 5-fluorouracil (5-FU), 8-azaguanine and cisplatin [102, 103]. Activation of organized mechanisms from detoxification systems such as DNA-repair and cytochrome P450 oxidases can also lead to multidrug resistance, as seen in P-gp and CYP3A coordinated induction [104]. Besides these, other systems include superoxide dismutase, catalase, glutathione peroxidase, and antioxidants. Induction of malignancy in cancers can result in mutant or non-functional p53; however, malignancy can also be a repercussion of chemotherapy. For example, fluctuation of ceramide levels or alteration of cell-cycle machinery may activate apoptotic checkpoints, thereby allowing cancer cells to avert apoptosis (Fig. 2) [105].

One of the most important genetic aspects influencing drug response is single nucleotide variants, however, insertions, deletions, repeats, and copy number variants can also have consequences in efficacy of therapy [106–108]. These mutations and variations are observed in genes responsible for the production of drug metabolizing enzymes, drug efflux genes, drug targets, DNA maintenance machinery, apoptotic machinery, and alleles related to the immune system [109–117]. DNA maintenance machinery reinstates genomic stability to maintain homeostasis and prevent cancer development. However, mutations and variations cause loss of normal function in DNA maintenance mechanisms, which leads to carcinogenesis, accelerated tumor evolution and resistance to drugs that attack DNA such as platins, nitrogen mustards, and chloroethyl nitrosoureas. The mechanism includes the formation of a DNA adduct that interferes with active DNA replication in cancer cells [118]. The generation of replication stress in a cell drives it towards apoptosis. DNA lesions, single-strand breaks or double-strand breaks are usually repaired by various DNA damage repair pathways such as base excision repair, nucleotide excision repair, homologous recombination, non-homologous end joining, mismatch repair, trans lesion synthesis and the Fanconi anemia pathway [119–122]. Epigenetic factors also have a vital role in various cancers. DNA methylation, one of the most common epigenetic alteration, which is carried out by DNA methyltransferases (DNMTs) that covalently attach a methyl group to cytosine triphosphate of the CpG islands in the genome [123, 124]. However, inhibition of DNMTs can cause the reversal of DNA methylation and revive the expression of the silenced genes [125]. Furthermore, other important histone modifications such as acetylation, phosphorylation, ubiquitination, sumoylation and adenosine di-phosphate (ADP) ribosylation can all lead to gene silencing. Cell differentiation and cell proliferation pathways such as mitogen-activated protein kinase (MAPK), Wnt, vascular endothelial growth factor (VEGF), p53 signaling, etc. are some of the most affected pathways due to aberrant epigenetics in respective genes [126, 127]. Additionally, micro RNAs (miRNAs) are a broadly conserved class of RNAs found mostly in intronic regions that are roughly 20–25 bases long polynucleotides and do not express any protein. The main function of miRNA is to downregulate gene expression at a post-transcriptional level, but it can also activate mRNA translation on rarer occasions. The network of miRNAs is so complex that each miRNA can regulate several different mRNAs and the same mRNA can be targeted by several different miRNAs as well [128]. The importance of miRNAs lies in the expression of the MDR efflux transporters of the ABC superfamily, since miRNAs regulates the post-transcriptional activity of MDR efflux transporters in several different tumors (Fig. 2) [129]. For instance, there has been a report of downregulation of ABCB1/MDR1 encoding for P-gp by several miRNAs such as miR-331-5p and miR-27a in lymphocytic and myeloid leukemia, miR-let-7 in ovarian cancer, miR-200c and miR-195 in breast cancer, miR-30a in gastric cancer and miR-9-3p in CML, thereby reversing the phenomenon of drug resistance [130–135]. Moreover, miR-326 regulation of ABCA2 in pediatric acute lymphoblastic leukemia has been reported to have an impact on drug resistance mechanisms. Along with, miRNAs can modulate the induction of apoptosis [136].

Drug-drug interactions (DDI) are observed commonly in patients suffering from cancer, due to multiple administrations of different drugs, adjuvants, and medications to treat additional co-morbidities [137]. A classic example of pharmacokinetic DDI is the absorption of tyrosine kinase inhibitors (TKIs). Altered intra-gastric pH values and the activity of intestinal enzymes as well as drug transporters influence TKI absorption substantially. Most of the TKIs are weak bases, protonated and most soluble in the acidic setting. Consequently, an increase in pH values, such as with proton pump inhibitors, greatly decreases their solubility [138, 139]. It has been reported that about 30% of cancer patients take proton pump inhibitors (PPIs), H_2_-antagonists or anti-acids to relieve gastro-esophageal reflux and dyspepsia symptoms. PPIs are some of the most commonly prescribed drugs along with TKIs [140, 141]. Apart from that, there can be synergistic, antagonistic, or additive responses as well.

### The ABC superfamily of transporters

**Fig. 3:**
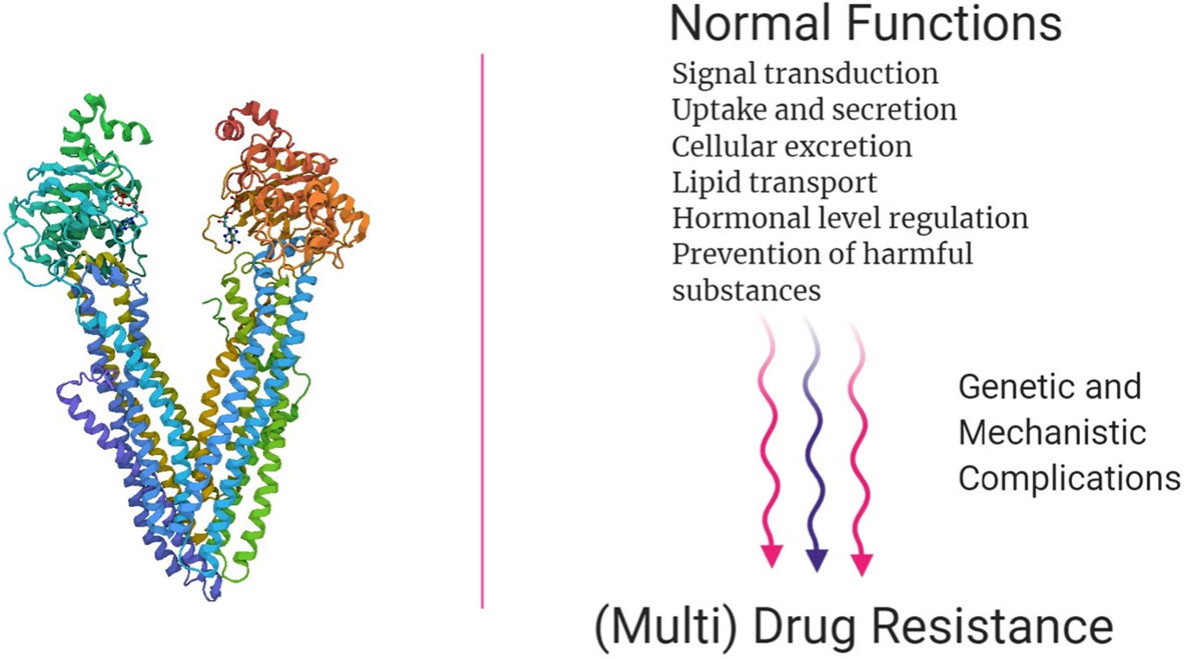
ABCB1 transporter (as a representative example of ABC superfamily of transporters). Normal functions of ABC transporters. (structure from RCSB PDB)

During the past years, various drug transporters pertaining to human cancers were identified and some of the latest are summarized in Table 2. **ABCB1**/MDR1 (also called P-gp) transporter was the first multi-drug transporter to be discovered as a surface phospho-glycoprotein that transports drugs and phospholipids across the membranes (Fig. 3). It is found on chromosome 7p21 and has 170 kDa of molecular weight [142, 143]. The structure of P-gp comprises of two homologous NBDs and two homologous TMDs with at least three sites for the binding of substrates/inhibitors. Interestingly the overexpression of P-gp alone confers drug-resistance to a huge number of neutral and cationic hydrophobic chemotherapeutic substrates including taxanes, epipodophyllotoxins, vinca alkaloids, anthracyclines, BCR-ABL TKIs, and epidermal growth factor receptor (EGFR) TKIs [101, 144–152].

**Table 2:** ABC transporters in human cancers

Name/ Gene name	Other Names	Genomic Location	Organ/tissue localization	MW (kDa)	Resistance conferred to	Modulators	References
MDR1/ ABCB1	P-gp, GP170, CLCS, ABC20	7p21	Blood-brain barrier, Bone marrow, Placenta, Gut mucosa, Liver, Kidney	170	Taxanes, Epipodophyllotoxins, Vinca Alkaloids, Anthracyclines, BCR-ABL TKIs, EGFR TKIs, ALK TKIs (Crizotinib, Ceritinib)	Sapitinib, Ibrutinib, RN486, Erlotinib, Lapatinib, Tariquidar, Elacridar, Zosuquidar	[101, 142–160]
BCRP/ ABCG2	ABCP, MXR1, CD338, ABC15	4q22.1	Placental syncytiotrophoblasts, Small intestine, Epithelial tissue of colon, Canalicular membrane in the liver, Microvessel endothelium of human brain, in the veins and blood vessels	72	Nucleoside analogs, Anthracyclines, Flavopiridols, Methotrexate, Methotrexate polyglutamates, E_2_17βG, Camptothecin-derived topoisomerase I inhibitors, GSK1070916, Tivantinib, Pevonedistat, Tozasertib, Barasertib	Erlotinib, Lapatinib, Icotinib	[153, 159, 161–176]
PRP/ ABCB6	DUH13, PSHK2, ABC14	2q35.5	Brain, Retina, Testis, Gall bladder, Intestine	93.8	Paclitaxel, 5-FU, Epirubicin, Cyclophosphamide, Daunorubicin, SN-38, Vincristine	Verteporfin, Tomatine HCl, Benzethonium chloride	[177–182]
MRP1/ ABCC1	GS-X, ABC29	16p13.1	Placenta, BBB, Lungs, Testis, Skeletal/Cardiac muscles, Kidney, Intestine	171.6	Anthracyclines, Vinca alkaloids, Epipodophyllotoxins, Camptothecins, GSH conjugates, Methotrexate, Mitoxantrone, Imatinib, Arsenite, Colchicine, Flutamide, Betulin, Saquinavir, Ritonavir, Indinavir	Everolimus, GSK1904529A, Rapamycin, Tipifarnib, TAK-733, Delavirdine, Indomethacin, Verapamil	[145, 183–194]
MRP2/ ABCC2	cMOAT, cMRP, ABC30	10q24.2	Placenta, BBB, Lungs, Kidney, Liver, Intestine	174.2	LTC4, E_2_17βG, GSH, Taxanes, Anthracyclines, Vinca alkaloids, Methotrexate, Mitoxantrone, Etoposide, Irinotecan, Cisplatin, SN-38, Saquinavir, Ritonavir, Lopinavir, Indinavir	Curcumin, Piperine, Rhinacanthin-C, Probenecid	[59, 195–202]
MRP3/ ABCC3	cMOAT2, MOATD, MLP2, ABC31	17q21.33	Placenta, Colon, Prostate, Kidney, Liver, Small intestine	169.3	Glutathione, Bile salts, LTC4, Glucuronide conjugates, DNP-SG, Vincristine	Fidaxomicin, Suramin, Lamivudine, Tenofovir	[203–208]
MRP4/ ABCC4	MOAT-B, ABC32	13q32.1	Blood, Pancreas, Adrenal gland, Prostate^a^, Kidney^a^, Liver^a^	149.5	cAMP^b^, cGMP^b^, Loop diuretics, Cephalosporins, Topotecan, Imatinib, PMEA, 6-MP, 6-TG, Methotrexate, Plant polyphenols, Resveratrol, Quercetin, Adefovir, Ganciclovir, Tenofovir, Zidovudine	Micafungin, Rofecoxib, Indomethacin, Verapamil	[194, 209–217]
MRP5/ ABCC5	SMRP, MOATC, ABC33	3q27.1	Brain, Testis, Skeletal/Cardiac Muscles	160.6	cAMP^b^, cGMP^b^, Folates, 6-MP, 5-FU, Methotrexate, Stavudine	Zaprinast, Benzbromarone, Sulfinpyrazone, Sildenafil, Sulfinpyrazone	[216, 218–222]
MRP6/ ABCC6	MLP1, MOAT-E, ABC34	16p13.11	Kidney, Liver	164.9	Cyclopentapeptide BQ123, Etoposide, Teniposide, Doxorubicin, Daunorubicin, LTC4, n-ethylmaleimide-glutathione	Indomethacin, Benzobromarone	[194, 223–225]
MRP7/ ABCC10	SIMRP7	6p21.1	Brain, Lungs, Testis, Blood, Prostate, Ovary, Kidney	161.6	Taxanes, Vinca alkaloids, 2′,3′-dideoxycytidine, AraC, PMEA, Epothilone B, LTC4, E_2_17βG	Nilotinib, Lapatinib, Tandutinib, PD-173074, Tariquidar	[211, 226–237]
MRP8/ ABCC11	EWWD, CFTR	16q12.1	Placenta^a^, Brain^a^, Liver^a^	154.3	DHEAS, E_1_S, E_2_17βG, LTC4, DNP-SG, AraC, 5-FU, Methotrexate, PMEA, 2′,3′ − dideoxycytidine, Adefovir		[194, 238–242]

^a^Indicates low levels of expression

^b^Indicates that inhibition of MRP4 results in impaired cAMP and cGMP transport

**ABCG2**/BCRP (Breast cancer resistance protein) is a 72 kDa MDR transporter with the gene located on chromosome 4q22.1, consisting of one TMD and one NBD, sometimes also called a “half transporter” [161, 162]. It gets activated upon homodimerization or oligomerization with itself or with other MDR transporters; a very similar mechanism to that of bacterial drug efflux transporters [153, 163, 243, 244]. It is one of the most widely distributed transporters and its expression is seen on the plasma membrane, highly expressed in placental syncytiotrophoblasts, the apical surface of small intestines, epithelial tissue of colon, canalicular membrane in the liver, microvessel endothelium of human brain and in the veins and blood vessels [164–167]. ABCG2 confers resistance to nucleoside analogs, anthracyclines, flavopiridols, methotrexate, organic dyes, and anionic conjugates, TKIs and camptothecin-derived topoisomerase I inhibitors [163, 168, 169].

**ABCB6**/PRP (P-gp related protein) is a 93.8 kDa protein with its gene located on chromosome 2q35.5. It is a mitochondrial transporter that can import heavy metals and regulate porphyrin biosynthesis and hence plays a role in cell cycle progression [177]. It is localized in mitochondria, ER and golgi apparatus. It has been reported that ABCB6 confers resistance to camptothecin and camptothecin-11 (CPT-11) in A549 lung cancer cells [178]. It also confers resistance to paclitaxel/FEC (5-FU, epirubicin, and cyclophosphamide) in breast cancer [179], daunorubicin in acute myeloid leukemia [180], and 5-FU, SN-38, and vincristine in Arsenic resistant KB-3-1 cells (KAS cells) [181].

**ABCC1**/MRP1 was first observed in anthracycline-resistant cell lines H69AR and HL60/Adr [183–185]. It is a 171.6 kDa protein with its gene located on chromosome 16p13.1. ABCC1 has three MSDs and two NBDs with an unusual MSD0 domain. It has a low degree of similarity with ABCB1 (15%), however, the resistance profile is quite comparable [186]. The normal function of MRP1 is xenobiotic detoxification of intermediates of phase II enzymatic reactions [245]. It confers resistance to drugs by ATP- and GSH-dependent export, however, it hydrolyzes ATP with a lower efficiency. ABCC1 has resistance against anthracyclines, vinca alkaloids, epipodophyllotoxins, camptothecins, methotrexate, and mitoxantrone [145, 187–189]. However, ABCC1 does not confer resistance to taxanes.

BCR-ABL TKIs- Breakpoint cluster regions- Abelson tyrosine kinase inhibitors; EGFR TKIs- Epidermal growth factor receptor TKIs; ALK TKIs- Anaplastic lymphoma kinase TKIs; E217βG- Estradiol 17 β-D glucuronide; 5-FU- 5-Fluorouracil; LTC4- Leukotriene C4; GSH- Glutathione; PMEA- 9-(2-phosphonyl methoxyethyl) adenine; 6-MP- 6-Mercaptopurine; 6-TG- 6-Thioguanine; cAMP- Cyclic adenosine monophosphate; cGMP- Cyclic guanosine monophosphate; DHEAS- Dehydroepiandrosterone 3-sulfate; DNP-SG- S-(2,4-dinitrophenyl) glutathione.

**ABCC2**/MRP2 gene is located on the chromosome 10q24.2. It encodes for MRP2 protein, which is found to be 174.2 kDa in molecular weight. It consists of two NBDs, two membrane spanning domains (MSDs), an NH_2_ terminal MSD0 with five transmembrane helices, and an intracellular linker segment L0. This kind of structural organization is also observed in MRP1, − 3, − 6 and − 7; while the MRP4, − 5, − 8 and − 9 do not have the MSD0 domain [185, 195, 246, 247]. MRP2 is also known as canalicular multi-specific organic anion transporter (cMOAT), named after its function of transporting amphipathic anionic conjugates of phase II conjugation reactions into the bile [245]. It is principally expressed at hepatocyte canalicular membrane, epithelial cells of the gall bladder, and in apical membranes of the proximal tubule in human kidneys [59, 196, 248]. MRP2 administers resistance against a wide spectrum of drugs like glutathione, glucuronates and sulfates [195]. It confers resistance to cisplatin as well [197].

**ABCC3**/MRP3 plays a crucial role in conferring multidrug resistance to cancer cells. It is a 169.3 kDa protein with gene located on chromosome 17q21.33 and it bears 58% structural similarity with MRP1 (highest similarity) with three TMDs and two NBDs [206, 207]. MRP3 is an organic anion transporter with a higher affinity for GSH conjugates and it may also play an important role in bile circulation. It provides resistance against leukotriene C4 (LTC4), S-(2,4-dinitrophenyl) glutathione (DNP-SG), glutathione sulfate and glucuronide conjugates [203]. It has been found that ABCC3 expression is correlated with doxorubicin resistance in Lung cancer patients [204, 205].

**ABCC4**/MRP4 is one of the shortest members of the ABC-superfamily of transporters; with only 1325 amino acids in its polypeptide sequence. The gene encoding MRP4 is located on chromosome 13q32.1 and the translated protein weighs about 149.4 kDa [209]. MRP4 has a typical structure containing two NBDs and two MSDs; and each MSD consists of six TMDs [216]. MRP4 further regulates the synthesis and efflux of prostaglandins and thereby play a role in inflammation [249]. Substrates of MRP4 include cyclic adenosine monophosphate (cAMP), cyclic guanosine monophosphate (cGMP), adefovir, ganciclovir, loop diuretics, cephalosporins, topotecan, PMEA, 6-mercaptopurine (6-MP), 6-thioguanine (6-TG), etc. [210–212, 214]. Recent updates added a few more natural products like plant polyphenols, resveratrol, and quercetin that are transported by MRP4 [213].

**ABCC5**/MRP5 weighs 160.6 kDa and the gene is found on the chromosome 3q27.1. This transporter is highly localized in brain, heart, skeletal muscles, and lungs [209, 220]. It is similar to MRP4, except, it is devoid of MSD0 and contains a 90 aminoacyl long hydrophilic extension in the sixth TMD [218–220]. MRP5 is an organic anionic transporter, which can transport nucleotides and nucleotide analogs like cAMP, cGMP, 6-MP, 5-FU, and antifolates like methotrexate [221].

**ABCC6**/MRP6 has a 41% structural similarity with MRP1 and is comprised of two NBDs and three MSDs with five, six and six TMDs, respectively. It is 164.9 kDa in molecular weight and its gene is located on chromosome 16p13.11 [223, 250]. It is mainly localized in kidney and liver and has low or miniscule levels in other tissues [223]. It functions as a calcium transporter to regulate tissue calcification. It confers resistance against cyclopentapeptide BQ123, low resistance against etoposide, teniposide, anthracyclines and cisplatin. It can also expel out glutathione (GSH) conjugates like LTC4 and n-ethylmaleimide-glutathione [224, 225].

**ABCC10**/MRP7 gene is found on chromosome 6p21.1 and is a 161.6 kDa protein. It consists of three MSDs and two NBDs [226, 251, 252]. It is found to be expressed in tissues like skin, testes, spleen, stomach, colon, kidney, heart, and brain (sparse proportions) [226]. Although, its expression in the pancreas, liver, placenta, and spleen is much higher [227]. MRP7 is a lipophilic anion transporter with functions in transporting GSH conjugates and glucuronides, and tissue detoxification. Substrates of MRP7 include glucuronides (17-β-D glucuronide) and the glutathione conjugate of LTC4 [228]. HEK293 cells with overexpression of ABCC10 confers resistance to taxanes, vinca alkaloids, 2′,3′-dideoxycytidine, 9-(2-phosphonyl methoxyethyl) adenine (PMEA), and epothilone B [211, 226, 228–235, 253].

**ABCC11**/MRP8 is another transporter similar to MRP4 and MRP5, holding two MSDs, two NBDs and 12 TMDs. It weighs 154.3 kDa and the gene is located on chromosome 16q12.1. It is broadly apportioned throughout the body with high levels in breast, brain, liver, placenta, and testes [238]. It is found that MRP8 is an important part of central and peripheral nervous system as it transports dehydroepiandrosterone 3-sulfate (DHEAS), a neuromodulatory steroid [239]. MRP8 is associated with cellular transport of DHEAS, E_1_S, estradiol 17 β-D glucuronide (E_2_17βG), some nucleotide analogs, lipophilic anions like LTC4 and DNP − SG, AraC, 5-FU, methotrexate, PMEA and 2′,3′ − dideoxycytidine [240–242].

In addition to ABC transporters, there are extracellular vesicles (EVs) present in the cells that can carry out drug efflux. EVs are about 30–1000 nm sized particles enclosed by a phospholipid bilayer, which cannot replicate [254, 255]. EVs were conventionally addressed as exosomes, microparticles, apoptotic bodies, microvesicles, etc. depending on their biogenesis, size, and content [256]. Interestingly, ABC transporters like P-gp, MRP1 and BCRP are associated with EVs [254, 257]. It is highly probable that the direction of these transporters might be reversed in some but not all EVs, such that there is an influx of drugs into the EVs. Influx of drugs into the EVs will lead to a decreased drug concentration inside the cell, resulting in drug resistance [257–259].

## Discussion

**Fig. 4:**
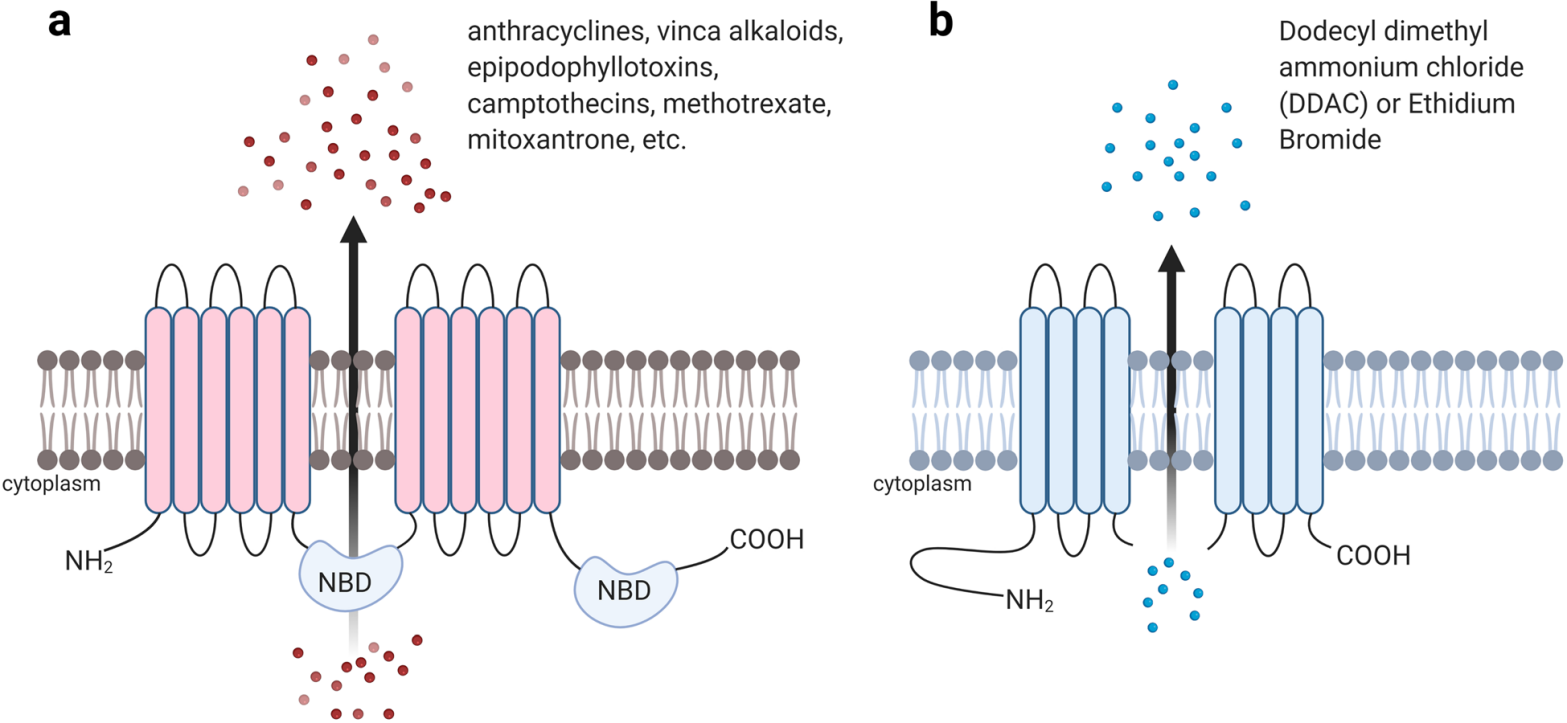
Comparative representation P-gp and EmrE. **a** ABCB1 (P-gp) transporter, one of the most frequently overexpressed transporters in human cancers. ABCB1 confers resistance to anthracyclines, vinca alkaloids, epipodophyllotoxins, camptothecins, methotrexate, mitoxantrone, etc. **b** EmrE transporter of *Escherichia coli*, belonging to SMR family of drug efflux transporters. EmrE confers resistance to dodecyl dimethyl ammonium chloride (DDAC) or Ethidium bromide

This review provides a brief overview and updates on the biochemistry and pharmacology of drug resistance in bacteria and cancer cells. Extensive research of over four decades has now culminated in the identification of thousands of ABC ATPases [260] and it is quite indisputable that all the three phyla of life share the ABC superfamily genes in some variation [55–57, 59, 261–268]. For instance, LmrA and EmrE found in bacterium *Lactococcus lactis* are structurally and functionally comparable to P-gp in humans (Fig. 4). The structure of MsbA of bacterial origin is also structurally comparable to inward facing conformation of mammalian P-gp [49, 269]. MDR in bacteria constitutes a huge, mutual reservoir of resistance determinants to most families of antimicrobial agents across numerous higher species, including humans. Modifications in cell membrane and its associated proteins lead to many causes of MDR in bacteria. Although, recent advances in imaging and organizational studies of MDR proteins have led us to better understand the molecular mechanisms of multidrug transport. The contribution of man-made activities like release of toxic chemicals into the ecosystem has built up an evolutionary survival pressure on bacteria. Both bacteria and cancer have similar ways to circumvent the phenomenon of cell death: a simpler way for bacteria is to swim away from the cytotoxic environment, which corresponds to metastasis in tumors. Another course of action is the formation of biofilms, where the bacterial colony creates a survivable surrounding, which corresponds to formation of altered tumor microenvironments including a vasculature. Although, these approaches of drug resistance are quite reversible in nature. Furthermore, while comparing the causalities of intrinsic or acquired resistance in bacteria and cancers (comparing Figs. 1 and 2), it is found that the basic idea behind the various mechanisms is supported by the DNA repair mechanisms. For instance, homologous recombination, DNA mismatch repairs, cell cycle regulation, etc. are very much similar mechanisms of MDR in both bacteria and cancer. These causalities lead to heritable resistances in both parties along with pre-existing genetic variations within the population and the origination of *de novo* mutations. The intrinsic or acquired resistance to drugs are more of a permanent nature as compared to previous reversible approaches [270]. Lambert et al. [270] discusses about stress-induced mutagenesis, which is usually advantageous to the whole population. The survival pressure exerted on bacteria promotes adaptive mutations. These adaptive mutations generally originate from point mutations, and in addition, external oxidative stress recurrently affects the accuracy of DNA transcription. Subsequently, it will lead to the production of mutant protein with a non-permanent alteration to the DNA template; also known as transcriptional mutagenesis [271]. However, in cancer, the occurrence is often, and it is referred to genetic instability. Conversely, it is safe to assume that genetic instability is an organized tactic to speed up adaptation which will finally be beneficial to malignancies.

Genetic heterogeneity of cancer cells in a single population is acquired by exposure to various chemotherapeutic drugs. Thus, in any cell population of a tumor mass already exposed to chemotherapy, there may be multiple mechanisms of MDR observed, which is termed as multifactorial MDR [101]. Additionally, the normal functions of the ABC transporters discussed above have a consequential effect on the pharmacokinetics of drugs through their absorption, distribution, metabolism, excretion, and toxicity. Inhibition of these transporters produces changes in the pharmacokinetics, toxicities, DDIs, and other complexities [272]. DDIs are preeminent in patients who have been prescribed drugs with a narrow therapeutic index and inherent toxicity, bringing about poor patient compliance and therapeutic decline. Since the activity of ABC transporters in MDR is not readily identified, it is necessary to develop a reliable discovery system. A reliable discovery system would help researchers determine the optimal combination of drug(s) and inhibitor(s), which in turn would aid clinicians in using the ABC transporters as clinical targets and cancer biomarkers.

## Data Availability

Not applicable.

## Abbreviations

5-FU: 5-Fluorouracil
6-MP: 6-Mercaptopurine
6-TG: 6-Thioguanine
ABC: ATP-binding cassette transporters
ADP: Adenosine di-phosphate
ALK: Anaplastic lymphoma kinase
ATP: Adenosine tri-phosphate
BCRP: Breast cancer resistance protein
cAMP: Cyclic adenosine mono-phosphate
cGMP: Cyclic guanosine mono-phosphate
DDAC: Dodecyl dimethyl ammonium chloride
DDI: Drug-drug interactions
DHEAS: Dehydroepiandrosterone 3-sulfate
DNA: Deoxyribonucleic acid
DNMT: DNA methyltransferases
DNP-SG: S-(2,4-dinitrophenyl) glutathione
E_2_-17βG: Estradiol 17 β-D glucuronide
EGFR: Epidermal growth factor receptor
ER: Endoplasmic reticulum
EVs: Extracellular vesicles
HGT: Horizontal gene transfer
ISCR: Insertion sequences-common regions
LTC4: Leukotriene C4
MAPK: Mitogen-activated protein kinase
MATE: Multi-antimicrobial extrusion
MDR: Multidrug resistance
MFP: Membrane fusion protein
MFS: Major facilitator superfamily
miRNA: Micro RNAs
MRSA: Methicillin-resistant *Staphylococcus aureus*
MSD: Membrane spanning domain
NBD: Nucleotide binding domain
OMF: Outer membrane factor
P-gp: P-glycoprotein
PMEA: 9-(2-Phosphonyl methoxyethyl) adenine
PPI: Proton pump inhibitors
RND: Resistance/nodulation/division
rRNA: Ribosomal ribonucleic acid
SMR: Small multidrug resistance
TKI: Tyrosine kinase inhibitors
TMD: Transmembrane domain
TMS: Transmembrane segments
VEGF: Vascular endothelial growth factor
WHO: World Health Organization
